# Evolution of Penicillin Non-susceptibility Among *Streptococcus pneumoniae* Isolates Recovered From Asymptomatic Carriage and Invasive Disease Over 25 years in Brazil, 1990–2014

**DOI:** 10.3389/fmicb.2019.00486

**Published:** 2019-03-14

**Authors:** Tatiana Castro Abreu Pinto, Felipe Piedade Gonçalves Neves, Aline Rosa Vianna Souza, Laura Maria Andrade Oliveira, Natália Silva Costa, Luciana Fundão Souza Castro, Cláudia Rezende de Vieira Mendonça-Souza, José Mauro Peralta, Lúcia Martins Teixeira

**Affiliations:** ^1^Instituto de Microbiologia Paulo de Goes, Universidade Federal do Rio de Janeiro, Rio de Janeiro, Brazil; ^2^Instituto Biomédico, Universidade Federal Fluminense, Niterói, Brazil; ^3^Faculdade de Medicina, Universidade Federal Fluminense, Niterói, Brazil

**Keywords:** *Streptococcus pneumoniae*, penicillin non-susceptibility, asymptomatic carriage, invasive pneumococcal disease, capsular type

## Abstract

*Streptococcus pneumoniae* is a major cause of community-acquired pneumonia and meningitis, and it is also found as a commensal, colonizing the human upper respiratory tract of a portion of the human population. Its polysaccharide capsule allows the recognition of more than 90 capsular types and represents the target of the currently available pneumococcal conjugate vaccines (PCVs), such as the 10-valent (PCV10) and the 13-valent (PCV13). Penicillin non-susceptible pneumococci (PNSP) have been listed as one of the current major antimicrobial-resistant pathogen threats. In Brazil, the emergence of PNSP was initially detected in the mid 1990s and PCV10 has been part of the National Immunization Program since 2010. Here, we investigated the distribution of capsular types and penicillin susceptibility profiles of 783 pneumococcal strains isolated in Brazil between 1990 and 2014 to assess the evolution of penicillin non-susceptibility among pneumococci associated with asymptomatic carriage and invasive pneumococcal disease (IPD). The most common serotypes among carriage isolates were 19F, 6B, 6C, 23F, and 14. Among IPD isolates, the most frequent types were 14, 3, 6B, 5, 19F, and 4. We detected 21 types exclusively associated with IPD isolates, whereas non-typeable (NT) isolates were only detected in carriage. Nearly half of the isolates belonged to PCV10 serotypes, which remarkably decreased in occurrence (by nearly 50%) after PCV10 introduction (2011–2014), while non-PCV10 serotypes increased. PNSP frequency and levels were much higher among carriage isolates, but PNSP belonging to PCV10 serotypes were more common in IPD. While the occurrence of PNSP has decreased significantly among IPD isolates since 2011, it kept increasing among carriage strains. Such a difference can be attributed to the serotypes that emerged in each clinical source after PCV10 usage. PNSP with multidrug resistance profiles that emerged within carriage isolates comprised mostly serotypes 6C and 35B, as well as NT isolates. In turn, penicillin-susceptible capsular types 3, 20, and 8 have risen among IPD. Overall, our results reinforce the relevance of PNSP surveillance over a long period of time to better understand the dynamics of antimicrobial resistance in response to PCV introduction and may also contribute to improve control measures toward drug-resistant pneumococci.

## Introduction

*Streptococcus pneumoniae*, or pneumococcus, is a leading cause of infections, such as pneumonia and meningitis, among children > 5 years old. In addition, this microorganism is also commonly found colonizing the human upper respiratory tract, a niche considered as its major reservoir and the main entry for the establishment of invasive pneumococcal disease (IPD) (Lynch and Zhanel, 2009; Weiser, 2010; Tan, 2012; Donkor, 2013).

This pathogen presents a polysaccharide capsule as the most important virulence factor (Bogaert et al., 2004; Kadioglu et al., 2008; Hyams et al., 2010). The pneumococcal capsule is antigenically diverse allowing the recognition of more than 90 serotypes (Bentley et al., 2006; Mostowy et al., 2017). In addition, the polysaccharide capsule is the basis of licensed vaccine formulations against pneumococcal disease, including the 7-valent pneumococcal conjugate vaccine (PCV7), the 10-valent PCV (PCV10), and the 13-valent PCV (PCV13) (WHO, 2012).

Penicillin non-susceptible pneumococci (PNSP) were recently listed as one of the most important antimicrobial-resistant threats worldwide (CDC, 2013; WHO, 2017). Increasing occurrence of PNSP has been detected since the first report in 1967 in Australia (Hansmann and Bullen, 1967; Castañeda et al., 1998; Appelbaum, 2002; Sadowy et al., 2010; Hackel et al., 2013; Kim et al., 2016). This characteristic seems to be more commonly associated with certain serotypes, such as serotype 14 and those included in serogroups 6, 19, and 23 (McGee et al., 2001; Lee et al., 2014). In Brazil, the emergence of PNSP was initially documented in the mid 1990s and it was initially attributed to the introduction of an internationally disseminated clone (namely ST156) expressing the capsular type 14 (Brandileone et al., 2006; Pinto et al., 2016).

Different measures can affect the epidemiology and evolution of PNSP isolates, including antibiotic therapy policies and the implementation of vaccines. However, such interventions may vary according to the geographical region (Guillemot et al., 1998; McCormick et al., 2003; Kim et al., 2016). Brazil is one of the 32 countries that have introduced PCV10 into the national immunization program, starting in 2010 (Brazil Ministry of Health, 2010). In turn, PCV13 has simultaneously replaced PCV7 in private immunization clinics. Thus, the aim of the present study was to investigate the distribution of capsular types and penicillin susceptibility profiles among pneumococcal isolates recovered from asymptomatic carriage and IPD over a period of 25 years in Brazil, comprising the periods before and after PCV introduction.

## Materials and Methods

### Bacterial Strains

A total of 783 peumococcal isolates were included in the study, comprising 355 isolates recovered from asymptomatic carriers (nasopharynx or oropharynx specimens) and 428 strains derived from IPD (blood or cerebrospinal fluid specimens). They were isolated from children and adults between 1990 and 2014 in five different cities (Campos dos Goytacazes, Niterói, Ribeirão Preto, Rio de Janeiro, and São Paulo) of Southeastern Brazil.

Isolates were recovered during surveillance studies or received from health institutions. Isolates obtained from cases of infection were recovered from clinical specimens taken as part of the standard patient care procedures and did not require ethical approval for their use. Carriage isolates were recovered from specimens collected during surveillance studies approved by ethics committees.

The isolates were previously subjected to phenotypic identification tests according to standard procedures (Spellerberg and Brandt, 2011), including observation of colony morphology and hemolysis on blood agar plates, cellular characteristics as observed after Gram stain, and catalase production, optochin susceptibility and bile-solubility testing.

### Determination of Capsular Types

The capsular types were determined by either multiplex PCR (Dias et al., 2007) or the standard Quellung reaction (Sørensen, 1993) with antisera provided by the *Streptococcus* Laboratory at the Centers for Disease Control and Prevention (CDC, Atlanta, GA, United States).

### Evaluation of Penicillin Susceptibility Profiles

Susceptibility to penicillin was evaluated according to the CLSI recommendations and interpretative criteria (CLSI, 2016). Minimal inhibitory concentrations (MICs) of penicillin were determined by either using the broth microdilution method or E-test^®^ strips (Oxoid, bioMérieux). All isolates showing penicillin MICs ≥ 0.12 μg/ml were classified as PNSP. In addition, isolates showing penicillin MICs ≥ 0.12 < 2 μg/ml were classified as pneumococci with reduced susceptibility to penicillin (PRSP), those with MICs ≥ 2 < 4 μg/ml were classified as penicillin-resistant pneumococci (PRP) and those with MICs ≥ 4 μg/ml were classified as high-level penicillin resistant pneumococci (HLPRP).

### Statistical Analyses

Distribution of pneumococcal capsular types and penicillin resistance rates and levels were analyzed by the Chi-square or Fisher’s exact tests using the software GraphPad Prism v5.0. *p*-Values < 0.05 were considered significant.

## Results

### Distribution of Capsular Types

Sixty capsular types, as well as 13 non-typeable (NT) isolates, were detected among 783 pneumococcal isolates. Thirty-nine serotypes and NT isolates were identified among the 355 carriage isolates, and 59 serotypes were detected among the 428 IPD isolates. Twenty-one capsular types were exclusively observed in IPD derived strains, while only one serotype (7B) as well as NT isolates were exclusively identified in carriage strains. Supplementary Table S1 shows the distribution of capsular types among all 783 pneumococcal strains according to the clinical source.

**FIGURE 1:**
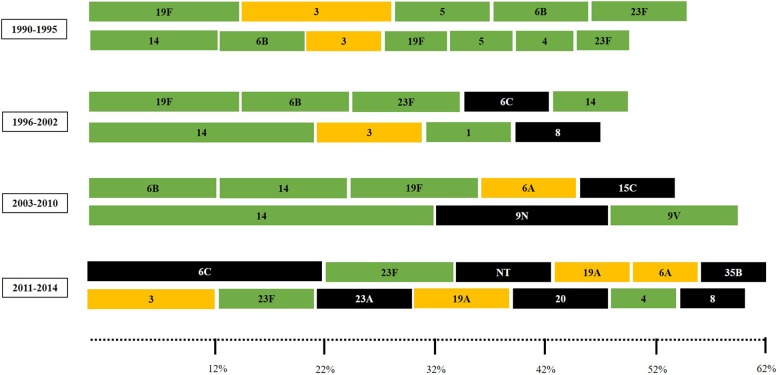
The most common pneumococcal capsular types (comprising 50 to 60% of the pneumococcal isolates) according to the clinical source and period of time. Carriage isolates (represented in the upper lines) included strains recovered from nasopharynx or oropharynx specimens while IPD (invasive pneumococcal disease) isolates (represented in the bottom lines) included strains recovered from blood or cerebrospinal fluid. Serotypes colored in green are included in the 10-valent pneumococcal conjugate vaccine (PCV); those colored in yellow are only included in the 13-valent PCV; and those colored in black are not included in any PCV currently available.

Overall, the most common serotypes were 14 (n = 86; 11%), 6B (n = 63; 8%), 19F (n = 62; 7.9%), 23F (n = 51; 6.5%), 3 and 6C (n = 40; 5.1% each), 6A (n = 26; 3.3%), and 5 (n = 25; 3.2%). These eight capsular types accounted for nearly half of the 783 strains. The most frequent serotypes among carriage strains were 19F (11.8%), 6B (9.6%), 6C (9%), 23F (8.7%), and 14 (8.2%); accounting for 47.3% of the isolates. In turn, the most common serotypes among IPD were 14 (13.3%), 3 (7.2%), 6B (6.5%), 5 (5.1%), 19F (5.1%) and 4 (4.7%), making up 41.9%. Distribution of serotypes fluctuated over time and a higher diversity of capsular types was detected in the late study period (Figure 1).

**FIGURE 2:**
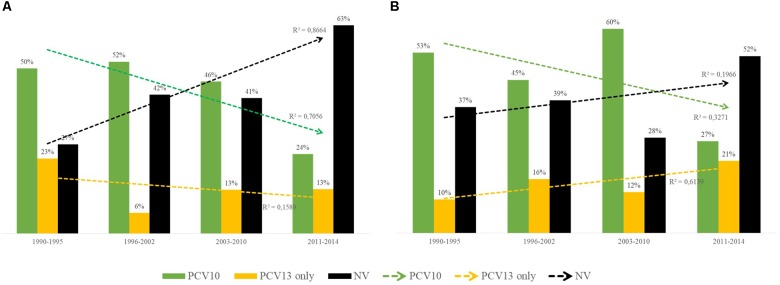
Distribution over time of capsular types included in the 10-valent pneumococcal conjugate vaccine (PCV10; in green), of those included only in the 13-valent pneumococcal conjugate vaccine (PCV13; in yellow) and of those not included in any PCV currently available [non-vaccine (NV), in black]. **(A)** Distribution among 355 *Streptococcus pneumoniae* isolates recovered from asymptomatic carriers. **(B)** Distribution among 428 *S. pneumoniae* isolates recovered from patients with IPD. 10-valent PCV includes serotypes 1, 4, 5, 6B, 7F, 9V, 14, 18C, 19F, and 23F; 13-valent PCV also includes 3, 6A, and 19A.

Nearly half of the 783 pneumococcal isolates belonged to PCV serotypes (Table 1 and Supplementary Table S1). Occurrence of PCV10 serotypes remarkably decreased during 2011–2014, while non-PCV10 serotypes, including non-vaccine (NV) serotypes and those exclusively covered by PCV13, increased in this same period (Figure 2). This trend was noted regardless of clinical source (p < 0.01). Of note, although detected in low numbers until 2010, all newly emerging non-PCV10 serotypes in the period 2011–2014 have been circulating in our setting since the early period of isolation included in the present study (1990s).

**Table 1:** Distribution of capsular types included in pneumococcal conjugate vaccines currently available among 783 *Streptococcus pneumoniae* isolates according to the clinical source.

Clinical source^a^	% (n) strains belonging to	
(n)	capsular types included in^b^	
	10-Valent PCV	13-Valent PCV
Carriage (355)	44.5 (158)	55.5 (197)
IPD (428)	50.5 (216)	61.9 (265)
All (783)	45.1 (353)	56.3 (441)

*^*a*^Carriage isolates included strains recovered from nasopharynx or oropharynx specimens while IPD (invasive pneumococcal disease) isolates included those recovered from blood or cerebrospinal fluid*.

*^*b*^PCV, pneumococcal conjugate vaccine; 10-valent PCV includes serotypes 1, 4, 5, 6B, 7F, 9V, 14, 18C, 19F, and 23F; 13-valent PCV also includes 3, 6A, and 19A*.

### Penicillin Susceptibility Profiles

Around 20% (176) of the 783 isolates were PNSP, showing penicillin MICs ranging from 0.12 to 8 μg/ml. Differences were noted regarding distribution of PRSP, PRP, and HLPRP between carriage and IPD, with significantly higher numbers and levels of penicillin resistance among carriage strains (Table 2; *p* < 0.05).

**Table 2:** Distribution of *Streptococcus pneumoniae* isolates with reduced susceptibility to penicillin (PRSP), resistant to penicillin (PRP), and high-level resistant to penicillin (HLPRP) according to the clinical source.

Clinical	PRSP%	PRP%	HLPRP%	MIC50	MIC90
source^*a*^	(N)	(N)	(N)	(μg/ml)	(μg/ml)
Carriage	23.9 (85)	3.7 (13)	5.6 (20)	0.06	1.5
IPD	11.4 (49)	0.5 (2)	1.6 (7)	0.03	0.12
All	17.1 (134)	1.9 (15)	3.4 (27)	0.03	0.5

*^*a*^Carriage isolates included those recovered from nasopharynx or oropharynx specimens while IPD (invasive pneumococcal disease) isolates included those recovered from blood or cerebrospinal fluid. Isolates showing penicillin MICs ≥ 0.12 < 2 μg/ml were classified as pneumococci with reduced susceptibility to penicillin (PRSP), those with MICs ≥ 2 < 4 μg/ml were classified as penicillin-resistant pneumococci (PRP) and those with MICs ≥ 4 μg/ml were classified as high-level penicillin resistant pneumococci (HLPRP)*.

Overall, PNSP were associated with 24 serotypes and NT isolates (Supplementary Table S1); eight serotypes (6A, 6B, 6C, 14, 19A, 19F, 23F, and 35B) and NT isolates were mostly associated with penicillin resistance (Table 3). These serotypes included six (6A, 6B, 6C, 14, 19F, and 23F) of the most frequently found among the 783 isolates investigated. In addition, four (6B, 14, 19F, and 23F) of them were PCV10 serotypes. Nevertheless, the most common PNSP serotypes varied according to the clinical source (Table 3). Of note, a much higher proportion of PNSP strains belonging to PCV10 serotypes was isolated from IPD (Table 4; *p* < 0.01).

**Table 3:** Distribution of *Streptococcus pneumoniae* isolates non-susceptible to penicillin (PNSP) among nine capsular types mostly associated with penicillin resistance, according to the clinical source.

Capsular type (n)	% PNSP (n)	Carriage^a^			IPD^a^		
		% PNSP	MIC50^b^	MIC90^b^	% PNSP	MIC50^b^	MIC90^b^
6A (26)	26.9 (7)	36.8	0.06	2	0	0.01	0.03
6B (63)	38.1 (24)	31.4	0.06	0.25	46.4	0.06	0.32
6C (40)	40 (16)	50	0.06	0.75	0	0.01	0.06
14 (86)	52.3 (45)	82.7	2	4	36.8	0.06	1.5
19A (22)	36.4 (8)	45.4	0.06	8	27.3	0.05	8
19F (62)	17.7 (11)	17.5	0.06	0.12	18.2	0.06	0.25
23F (51)	49 (25)	61.3	0.12	0.25	30	0.06	0.25
35B (7)	57.1 (4)	66.7	1	4	0	0.01	0.01
NT (13)	61.5 (8)	61.5	0.19	4	0	NA	NA

*^*a*^Carriage isolates included strains recovered from nasopharynx or oropharynx specimens while IPD (invasive pneumococcal disease) isolates included those recovered from blood or cerebrospinal fluid*.

*^*b*^μg/ml. NA, not applicable since no strain belonging to such serotype was detected. Strains showing penicillin MICs ≥ 0.12 μg/ml were classified as penicillin non-susceptible pneumococci (PNSP). Serotypes comprised by PCV10 are highlighted in green, those included only in PCV13 are highlighted in yellow, those not included in any PCV currently available are not colored*.

**Table 4:** Distribution of capsular types included in pneumococcal conjugate vaccines currently available among 176 *Streptococcus pneumoniae* isolates non-susceptible to penicillin (PNSP) according to the clinical source.

Clinical source^a^	% (n) of isolates belonging to	
(n)	capsular types included in^b^	
	10-Valent PCV	13-Valent PCV
Carriage (118)	53.4 (63)	63.5 (75)
IPD (58)	87.9 (51)	93.1 (54)
All (176)	45.1 (353)	56.3 (441)

*^*a*^Carriage isolates included strains recovered from nasopharynx or oropharynx specimens while IPD (invasive pneumococcal disease) isolates included those recovered from blood or cerebrospinal fluid*.

*^*b*^PCV, pneumococcal conjugate vaccine; 10-valent PCV includes serotypes 1, 4, 5, 6B, 7F, 9V, 14, 18C, 19F, and 23F; 13-valent PCV also includes 3, 6A, and 19A*.

**FIGURE 3:**
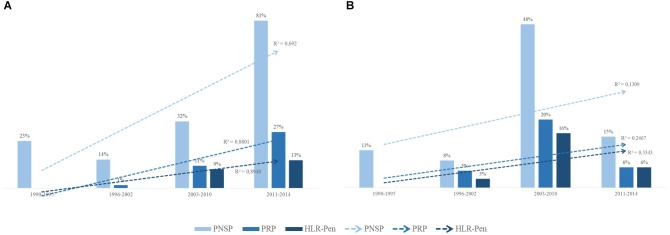
Distribution over time of pneumococci with reduced susceptibility to penicillin (PRSP), penicillin-resistant pneumococci (PRP), and high-level penicillin resistant pneumococci (HLPRP). **(A)** Distribution among 355 *S. pneumoniae* isolates recovered from asymptomatic carriers. **(B)** Distribution among 428 *S. pneumoniae* isolates recovered from patients with IPD. Isolates showing penicillin MICs ≥ 0.12 < 2 μg/ml were classified as PRSP, those with MICs ≥ 2 < 4 μg/ml were classified as PRP and those with MIC ≥ 4 μg/ml were classified as HLPRP.

PRSP, PRP and HLPRP showed an increasing trend during the study period among carriage strains (Figure 3A and Table 5; *p* < 0.01). Regarding IPD, this increasing trend was observed only until 2010; between 2011 and 2014, PNSP numbers and levels significantly decreased (Figure 3B and Table 5; *p* < 0.01).

**Table 5:** Distribution of penicillin minimum inhibitory concentration (MIC) levels among *Streptococcus pneumoniae*, according to the period of time and clinical source.

Clinical source^a^	MIC50 (μg/ml)				MIC90 (μg/ml)			
	1990–1995	1996–2002	2003–2010	2011–2014	1990–1995	1996–2002	2003–2010	2011–2014
Carriage	0.03	0.06	0.03	0.50	0.25	0.12	2	4
IPD	0.03	0.06	0.09	0.01	0.12	0.12	4	0.12

*^*a*^Carriage isolates included strains recovered from nasopharynx or oropharynx specimens while IPD (invasive pneumococcal disease) isolates included those recovered from blood or cerebrospinal fluid*.

**FIGURE 4:**
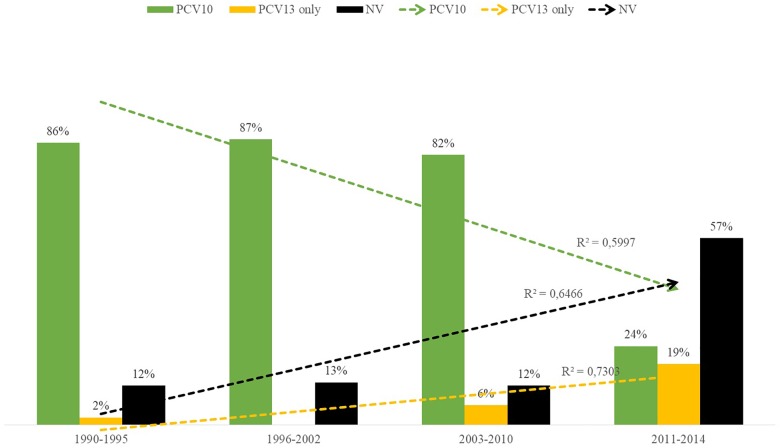
Distribution over time of capsular types included in the 10-valent pneumococcal conjugate vaccine (PCV10; in green), of those included only in the 13-valent pneumococcal conjugate vaccine (PCV13; in yellow) and of those not included in any PCV currently available (in black) among penicillin non-susceptible pneumococci (PNSP).

**FIGURE 5:**
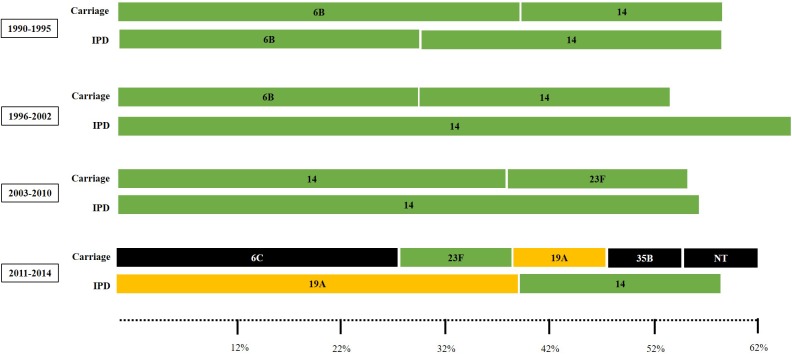
Sets of the most common pneumococcal capsular types associated with penicillin non-susceptibility (comprising 50 to 60% of the pneumococcal isolates) according to the clinical source and period of time. Carriage isolates included those recovered from nasopharynx or oropharynx specimens while IPD isolates included those recovered from blood or cerebrospinal fluid. Serotypes colored in green are included in the 10-valent pneumococcal conjugate vaccine (PCV); those colored in yellow are only included in the 13-valent PCV; and those colored in black are not included in any PCV currently available.

Distribution of PNSP serotypes also varied according to the study period. Overall, PNSP belonging to PCV10 serotypes showed a decreasing trend, while PNSP associated with non-PCV10 serotypes showed an increasing trend (Figure 4; *p* < 0.01). However, the most frequent serotypes in each period varied according to the clinical source. In addition, a higher diversity of serotypes was associated with PNSP isolated in the late period (Figure 5).

## Discussion

Differences in the distribution of pneumococcal serotypes between carriage and IPD isolates were observed. Some serotypes, including 3, 4, and 5, were exclusively detected among IPD cases. Previous studies have shown that certain capsular types are more prone to cause IPD while others are well-adapted to nasopharynx colonization (Bender et al., 2008; Weiser, 2010; Weinberger et al., 2011). Pneumococcal strains lacking the polysaccharide capsule (NT isolates), for example, are believed to be less virulent (Sharma et al., 2013). Accordingly, NT isolates were only identified among pneumococcal isolates recovered from asymptomatic carriage. On the other hand, a group of serotypes seems to be highly versatile, being frequently found in both carriage and IPD. In this study, three capsular types were frequently found regardless of clinical source, including 6B, 14, and 19F. Indeed, these serotypes are known to be common among carriage and IPD worldwide before PCV introduction (Hausdorff, 2007; Weinberger et al., 2011; Song et al., 2013).

Nearly 20% of all the isolates were PNSP, which is in accordance with previous data from Brazilian studies (Neves et al., 2013; Mott et al., 2014; dos Santos et al., 2015). However, differences on the distribution of penicillin resistance were also noted when carriage and IPD isolates were compared. PNSP occurrence, as well as penicillin MIC levels, were higher among carriage isolates. Indeed, certain serotypes almost exclusively found in IPD, such as serotype 3, were fully susceptible to penicillin. Several studies have shown that pneumococcal serotypes commonly found in carriage are more frequently associated with antimicrobial resistance than those exclusively found in IPD isolates (Weiser, 2010; Song et al., 2013; Zhou et al., 2015; Kim et al., 2016; Neves et al., 2017). This observation may be due, at least in part, to the fact that the human nasopharynx, in contrast to blood or cerebrospinal fluid, is a highly populated niche where genetic exchange among bacteria occurs and, thus, emergence of antimicrobial resistance traits can be favored (Andam and Hanage, 2015; Kim et al., 2016).

Although fluctuations on the occurrence of serotypes over time can happen naturally and should be carefully evaluated, our results suggest that the introduction of PCV7 and PCV13 in 2001 and 2010, respectively, did not seem to have affected pneumococcal epidemiology regarding serotype and PNSP distribution in our setting. This might be due, at least in part, to the fact that these PCVs were made available only in private clinics in Brazil. Indeed, usage of PCV7 and PCV13 in Brazil is very low due to their high cost (Brazil Ministry of Health, 2006; Medeiros et al., 2017; Neves et al., 2017). On the other hand, according to previous studies conducted in Brazil (dos Santos et al., 2013; Medeiros et al., 2017; Neves et al., 2018), our results suggest an important impact on serotype replacement after the implementation of PCV10. PCV10 serotypes showed a decreasing trend over time, especially in the late study period (2011–2014). In parallel, occurrence of non-PCV10 serotypes increased over time, surpassing the numbers of PCV10 serotypes in both carriage and IPD between 2011 and 2014. Similar observations have been made in other countries where PCV10 was routinely adopted, such as the Netherlands, Mozambique and Finland (Knol et al., 2015; Nhantumbo et al., 2017; Sihvonen et al., 2017).

Among the non-PCV10 serotypes emerging after PCV10 introduction, serotype 19A was an important serotype associated with both carriage and IPD. Although emergence of this serotype after PCV7 introduction in certain high-income countries is a well-established fact (Isaacman et al., 2010; Isturiz et al., 2017), serotype 19A emergence after PCV10 introduction in Brazil still seems to be a contradictory issue. While certain studies reveal that occurrence of this serotype has not significantly changed (Medeiros et al., 2017; Neves et al., 2018), others report an increasing rate (Cassiolato et al., 2018; Christophe et al., 2018). We also observed that serotypes 3, 8, 20, and 23A emerged among isolates from IPD cases, whereas serotypes 6C, 35B, and NT isolates were more commonly associated with asymptomatic carriage. Emergence of serotype 6C in carriage and of serotypes 3 and 8 in IPD after PCV10 implementation in Brazil has been recently described (Medeiros et al., 2017; Christophe et al., 2018; Neves et al., 2018). Of note, all these emerging non-PCV10 serotypes have been circulating in our setting since the 1990s, reinforcing the possibility of serotype replacement phenomenon.

Moreover, while PNSP numbers and levels decreased significantly in the late period of the present study (2011–2014) among IPD isolates, they kept increasing among isolates from carriage. Accordingly, many studies have reported lower frequencies and levels of PNSP among IPD isolates after PCV10 introduction in Brazil (dos Santos et al., 2013; Medeiros et al., 2017). In turn, antimicrobial resistance levels among pneumococcal isolates from asymptomatic carriage have been increasing despite of vaccination. Recently, Neves et al. (2018) have suggested that this is probably due to the emergence of multidrug resistant lineages belonging to non-PCV10 serotypes, such as the serotype 6C-CC386, among carriage isolates. On the other hand, serotypes emerging among IPD isolates after PCV10 introduction, such as 3, 8 and 20, were shown to be fully susceptible to penicillin. These observations suggest that the PCV10 impact on the reduction of PNSP occurrence and level might be more relevant for IPD than for carriage. This suggestion can also be supported by the observation that, before PCV10 introduction, PNSP isolates recovered from IPD were almost completely represented by PCV10 serotypes (nearly 90%), while only half of PNSP strains recovered from asymptomatic carriage comprised PCV10 serotypes.

Penicillin non-susceptible pneumococci have been listed as one of the major antimicrobial resistance threats among bacterial pathogens (CDC, 2013; WHO, 2017). Although they represent a global public health threat, occurrence and epidemiology of PNSP vary according to the geographic region. Taken our results into consideration, from the mid 1990s until 2010, serotype 14 played a major role in the dispersion of penicillin non-susceptibility, especially among IPD isolates. Indeed, it was previously shown that an internationally disseminated clone belonging to this serotype (namely ST156), which was also frequently associated with IPD worldwide, was the main reason for PNSP emergence in Brazil in the pre-vaccination era (Barroso et al., 2012; Pinto et al., 2016). After 2010, however, this scenario has changed and a more diversified panel of serotypes has been associated with penicillin non-susceptibility, regardless of clinical source. Among IPD isolates specifically, serotype 19A PNSP emerged significantly, surpassing the previous number of serotype 14 PNSP isolates.

Major limitations of this study are related to the characteristics of the population included. It is known that age of individuals is an important feature and may have an influence on serotype distribution. However, we were not able to assess this issue in detail since information was not available for a large proportion of the isolates analyzed, although we estimate from available data that most of strains were recovered from children. In addition, although Brazil is a country with continental dimensions and, thus, might present discrepancies between regions, the Southeastern region, represented here by five different cities, is the most populated one. According to the last official demographic survey conducted in Brazil (Instituto Brasileiro de Geografia e Estatística [IBGE], 2010), population living in the Southeastern region accounted for nearly half of the whole Brazilian population. Moreover, this region can be considered as representative of the ethnic, social, and economic diversity of the Brazilian population due to the historic high flow of domestic in-migration.

Penicillin non-susceptible pneumococci evolution can be driven by different interventions such as antibiotic therapy policies and vaccine implementation (Guillemot et al., 1998; McCormick et al., 2003; Kim et al., 2016). These aspects usually differ by country; for example, Brazil is one of the 32 countries that have adopted PCV10 in the national immunization program instead of PCV7/PCV13, adopted by other 98 countries (Brazil Ministry of Health, 2010). Therefore, gathering information on PNSP epidemiology over a long period of time can contribute to a better understanding of their evolution and the impact of different vaccination strategies. Overall, our results show the emergence of non-PCV10 serotypes after 2010 in Brazil and the emergence and spread of PNSP associated with carriage. On the other hand, PCV10 has been effective in decreasing PNSP rates and levels among IPD isolates, but it has not avoided serotype replacement. These results reinforce the need of continuous surveillance of PNSP in the post-vaccine introduction era and may contribute to the development of more effective measures to control the spread of drug-resistant pneumococci.

## Author Contributions

JP and LT coordinated the study. TP, LT, and JP contributed to the conception and design of the work. TP, FN, AS, LO, NC, CM-S, and LC performed the experiments and analyzed the data. LO performed the statistical analyses. TP, LO, JP, and LT wrote the manuscript. All authors revised and approved the final version of the manuscript.

## Conflict of Interest Statement

The authors declare that the research was conducted in the absence of any commercial or financial relationships that could be construed as a potential conflict of interest.

## References

[B1] Andam C. P. Hanage W. P. (2015). Mechanisms of genome evolution of *Streptococcus*. *Infect. Gent. Evol.* 33 334–342. 10.1016/j.meegid.2014.11.007 25461843 PMC4430445

[B2] Appelbaum P. C. (2002). Resistance among *Streptococcus pneumoniae*: implications for drug selection. *Clin. Infect. Dis.* 34 1613–1620. 10.1086/340400 12032897

[B3] Barroso D. E. Godoy D. Castiñeiras T. M. Tulenko M. M. Rebelo M. C. Harrison L. H. (2012). β-Lactam resistance, serotype distribution, and genotypes of meningitis-causing *Streptococcus pneumoniae*, Rio de Janeiro, Brazil. *Pediatr. Infect. Dis J.* 31 30–36. 10.1097/INF.0b013e31822f8a92 21860337 PMC4745886

[B4] Bender J. M. Ampofo K. Korgenski K. Daly J. Pavia A. T. Mason E. O. (2008). Pneumococcal necrotizing pneumonia in Utah: does serotype matter? *Clin. Infect. Dis.* 46 1346–1352. 10.1086/586747 18419434 PMC3673544

[B5] Bentley S. D. Mavroidi A. Saunders D. Rabbinowitsch E. Collins M. (2006). Genetic analysis of the capsular biosynthetic locus from all 90 pneumococcal serotypes. *PLoS Genet.* 2:e31. 10.1371/journal.pgen.0020031 16532061 PMC1391919

[B6] Bogaert D. de Groot R. Hermans P. W. M. (2004). *Streptococcus pneumoniae* colonization: the key to pneumococcal disease. *Lancet Infect. Dis.* 4 144–154. 10.1016/S1473-3099(04)00938-7 14998500

[B7] Brandileone M. C. C. Casagrande S. T. Guerra M. L. Zanella R. C. Andrade A. L. Di Fabio J. L. (2006). Increase in numbers of beta-lactam-resistant invasive *Streptococcus pneumoniae* in Brazil and the impact of conjugate vaccine coverage. *J. Med. Microbiol.* 55 567–574. 10.1099/jmm.0.46387-0 16585644

[B8] Brazil Ministry of Health (2006). *Manual dos Centros de Referência para Imunobiológicos Especiais 3^a^ı Edição.* Available at: http://bvsms.saude.gov.br/bvs/publicacoes/manual_centro_referencia_imunobiologicos.pdf

[B9] Brazil Ministry of Health (2010). *Introdução da Vacina Pneumocócica 10-Valente (Conjugada) no Calendário Básico de Vacinação da Criança.* Available at: http://www.sgc.goias.gov.br/upload/links/arq_723_infotec.pdf

[B10] Cassiolato A. P. Almeida S. C. G. Andrade A. L. Minamisava R. Brandileone M. C. C. (2018). Expansion of the multidrug-resistant clonal complex 320 among invasive *Streptococcus pneumoniae* serotype 19A after the introduction of a ten-valent pneumococcal conjugate vaccine in Brazil. *PLoS One* 13:e0208211. 10.1371/journal.pone.0208211 30496296 PMC6264150

[B11] Castañeda E. Peñuela I. Vela M. C. Thomaz A. (1998). Colombian pneumococcal study group Penicillin-resistant *Streptococcus pneumoniae* in Colombia: presence of international epidemic clone. *Microb. Drug Resist.* 4 233–239. 10.1089/mdr.1998.4.233 9818975

[B12] CDC (2013). *Antibiotic Resistance Threats in the United States, 2013.* Atlanta, GA: Centers for Disease Control and Prevention.

[B13] Christophe B. L. Mott M. da Cunha G. Caierão J. D. Azevedo P. Dias C. (2018). Characterization of *Streptococcus pneumoniae* isolates from invasive disease in adults following the introduction of PCV10 in Brazil. *J. Med. Microbiol.* 10.1099/jmm.0.000717[Epub ahead of print]. 29533176

[B14] CLSI (2016). *Performance Standards for Antimicrobial Susceptibility Testing. Twenty-Sixth Informational Supplement M100-S26.* Wayne, PA: Clinical and Laboratory Standards Institute.

[B15] Dias C. A. Teixeira L. M. Carvalho M. G. Beall B. (2007). Sequential multiplex PCR for determining capsular serotypes of pneumococci recovered from Brazilian children. *J. Med. Microbiol.* 56 1185–1188. 10.1099/jmm.0.47347-0 17761481

[B16] Donkor E. S. (2013). Molecular typing of the pneumococcus and its application in epidemiology in sub-Saharan Africa. *Front. Cell Infect. Microbiol.* 3:12. 10.3389/fcimb.2013.00012 23503978 PMC3596783

[B17] dos Santos M. S. Azevedo J. Menezes A. P. O. Cordeiro S. M. Escobar E. C. Lima J. B. (2015). Temporal trends and clonal diversity of penicillin non-susceptible pneumococci from meningitis cases from 1996 to 2012, in Salvador, Brazil. *BMC Infect. Dis.* 15:302. 10.1186/s12879-015-1049-y 26223380 PMC4520018

[B18] dos Santos S. R. Passadore L. F. Takagi E. H. Fujii C. M. Yoshioka C. R. Gilio A. E. (2013). Serotype distribution of *Streptococcus pneumoniae* isolated from patients with invasive pneumococcal disease in Brazil before and after ten-pneumococcal conjugate vaccine implementation. *Vaccine* 31 6150–6154. 10.1016/j.vaccine.2013.05.042 23747454

[B19] Guillemot D. Carbon C. Balkau B. Geslin P. Lecoeur H. Vauzelle-Kervroëdan F. (1998). Low dosage and long treatment duration of beta-lactam: risk factors for carriage of penicillin-resistant *Streptococcus pneumoniae*. *JAMA* 279 365–370. 10.1001/jama.279.5.365 9459469

[B20] Hackel M. Lascols C. Bouchillon B. Morgenstern D. Purdy J. (2013). Serotype prevalence and antibiotic resistance in *Streptococcus pneumoniae* clinical isolates among global populations. *Vaccine* 31 4881–4887. 10.1016/j.vaccine.2013.07.054 23928466

[B21] Hansmann D. Bullen M. M. (1967). A resistant pneumococcus. *Lancet* 2 264–265. 10.1016/S0140-6736(67)92346-X

[B22] Hausdorff W. P. (2007). The roles of pneumococcal serotypes 1 and 5 in paediatric invasive disease. *Vaccine* 25 2406–2412. 10.1016/j.vaccine.2006.09.009 17055620

[B23] Hyams C. Camberlein E. Cohen J. M. Bax K. Brown J. S. (2010). The *Streptococcus pneumoniae* capsule inhibits complement activity and neutrophil phagocytosis by multiple mechanisms. *Infect. Immun.* 78 704–715. 10.1128/IAI.00881-09 19948837 PMC2812187

[B24] Instituto Brasileiro de Geografia e Estatística [IBGE] (2010). *Censo 2010.* Available at https://censo2010.ibge.gov.br/.

[B25] Isaacman D. J. McIntosh E. D. Reinert R. R. (2010). Burden of invasive pneumococcal disease and serotype distribution among *Streptococcus pneumoniae* isolates in young children in Europe: impact of the 7-valent pneumococcal conjugate vaccine and considerations for future conjugate vaccines. *Int. J. Infect. Dis.* 14 197–209. 10.1016/j.ijid.2009.05.010 19700359

[B26] Isturiz R. Sings H. L. Hilton B. Arguedas A. Reinert R. R. Jodar L. (2017). *Streptococcus pneumoniae* serotype 19A: worldwide epidemiology. *Expert. Rev. Vaccines* 16 1007–1027. 10.1080/14760584.2017.1362339 28783380

[B27] Kadioglu A. Weiser J. N. Paton J. C. Andrew P. W. (2008). The role of *Streptococcus pneumoniae* virulence factors in host respiratory colonization and disease. *Nat. Rev. Microbiol.* 6 288–301. 10.1038/nrmicro1871 18340341

[B28] Kim L. McGee L. Tomczyk S. Beall B. (2016). Biological and epidemiological features of antibiotic-resistant *Streptococcus pneumoniae* in pre- and post-conjugate vaccine eras: a United States perspective. *Clin. Microbiol. Rev.* 29 525–552. 10.1128/CMR.00058-15 27076637 PMC4861989

[B29] Knol M. J. Wagenvoort G. H. Sanders E. A. Elberse K. Vlaminckx B. J. de Melker H. E. (2015). Invasive pneumococcal disease 3 years after introduction of 10-valent pneumococcal conjugate vaccine, the Netherlands. *Emerg. Infect. Dis.* 21 2040–2044. 10.3201/eid2111.140780 26488415 PMC4622227

[B30] Lee G. M. Kleinman K. Pelton S. I. Hanage W. Huang S. S. Lakoma M. (2014). Impact of 13-valent pneumococcal conjugate vaccination on *Streptococcus pneumoniae* carriage in young children in Massachusetts. *J. Pediatric. Infect. Dis. Soc.* 3 23–32. 10.1093/jpids/pit057 24567842 PMC3933044

[B31] Lynch J. P. Zhanel G. G. (2009). *Streptococcus pneumoniae:* epidemiology, risk factors, and strategies for prevention. *Semin. Respir. Crit. Care Med.* 30 189–209. 10.1055/s-0029-1202938 19296419

[B32] McCormick A. W. Whitney C. G. Farley M. M. Lynfield R. Harrison L. H. Bennett N. M. (2003). Geographic diversity and temporal trends of antimicrobial resistance in *Streptococcus pneumoniae* in the United States. *Nat. Med.* 9 424–430. 10.1038/nm839 12627227

[B33] McGee L. Wang H. Wasas A. Huebner R. Chen M. Klugman K. P. (2001). Prevalence of serotypes and molecular epidemiology of *Streptococcus pneumoniae* strains isolated from children in Beijing, China: identification of two novel multiply-resistant clones. *Microb. Drug Resist.* 7 55–63. 10.1089/107662901750152800 11310804

[B34] Medeiros M. I. C. Almeida S. C. G. Guerra M. L. L. S. da Silva P. Carneiro A. M. M. de Andrade D. (2017). Distribution of *Streptococcus pneumoniae* serotypes in the northeast macro-region of São Paulo state/Brazil after the introduction of conjugate vaccine. *BMC Infect. Dis.* 17:590. 10.1186/s12879-017-2696-y 28841854 PMC5574098

[B35] Mostowy R. J. Croucher N. J. De Maio N. Chewapreecha C. Salter S. J. Turner P. (2017). Pneumococcal capsule synthesis locus as evolutionary hotspot with potential to generate novel serotypes by recombination. *Mol. Biol. Evol.* 34 2537–2554. 10.1093/molbev/msx173 28595308 PMC5850285

[B36] Mott M. Caierão J. Rosa Da Cunha G. Rodrigues P. L. R. Matusiak R. Pilger O. K. R. (2014). Susceptibility profiles and correlation with pneumococcal serotypes soon after implementation of the 10-valent pneumococcal conjugate vaccine in Brazil. *Int. J. Infect. Dis.* 20 47–51. 10.1016/j.ijid.2013.11.009 24389158

[B37] Neves F. P. Pinto T. C. Corrêa M. A. Dos Anjos Barreto R. De Souza G. Moreira L. (2013). Nasopharyngeal carriage, serotype distribution and antimicrobial resistance of *Streptococcus pneumoniae* among children from Brazil before the introduction of the 10-valent conjugate vaccine. *BMC Infect. Dis.* 13:318. 10.1186/1471-2334-13-318 23849314 PMC3718621

[B38] Neves F. P. G. Cardoso N. T. Snyder R. E. Marlow M. A. Cardoso C. A. A. Teixeira L. M. (2017). Pneumococcal carriage among children after four years of routine 10-valent pneumococcal conjugate vaccine use in Brazil: the emergence of multidrug resistant serotype 6C. *Vaccine* 35 2794–2800. 10.1016/j.vaccine.2017.04.019 28431817

[B39] Neves F. P. G. Cardoso N. T. Souza A. R. V. Snyder R. E. Marlow M. M. Pinto T. C. A. (2018). Population structure of *Streptococcus pneumoniae* colonizing children before and after universal use of pneumococcal conjugate vaccines in Brazil: emergence and expansion of the MDR serotype 6C-CC386 lineage. *J. Antimicrob. Chemother.* 73 1206–1212. 10.1093/jac/dky001 29401243

[B40] Nhantumbo A. A. Weldegebriel G. Katsande R. de Gouveia L. Comé C. E. Cuco A. Z. (2017). Surveillance of impact of PCV-10 vaccine on pneumococcal meningitis in Mozambique, 2013 - 2015. *PLoS One* 12:e0177746. 10.1371/journal.pone.0177746 28604773 PMC5467806

[B41] Pinto T. C. Kegele F. C. Dias C. A. Barros R. R. Peralta J. M. Merquior V. L. (2016). *Streptococcus pneumoniae* serotypes 9 and 14 circulating in Brazil over a 23-year period prior to introduction of the 10-valent pneumococcal conjugate vaccine: role of international clones in the evolution of antimicrobial resistance and description of a novel genotype. *Antimicrob. Agents Chemother.* 60 6664–6672. 10.1128/AAC.00673-16 27572394 PMC5075071

[B42] Sadowy E. Kuch A. Gniadkowski M. Hryniewicz W. (2010). Expansion and evolution of the *Streptococcus pneumoniae* Spain9V-ST156 clonal complex in Poland. *Antimicrob. Agents Chemother.* 54 1720–1727. 10.1128/AAC.01340-09 20194703 PMC2863602

[B43] Sharma D. Baughman W. Holst A. Thomas S. Jackson D. da Gloria Carvalho M. (2013). Pneumococcal carriage and invasive disease in children before introduction of the 13-valent conjugate vaccine: comparison with the era before 7-valent conjugate vaccine. *Pediatr. Infect. Dis. J.* 32 45–53. 10.1097/INF.0b013e3182788fdd 23080290

[B44] Sihvonen R. Siira L. Toropainen M. Kuusela P. Pätäri-Sampo A. (2017). *Streptococcus pneumoniae* antimicrobial resistance decreased in the Helsinki metropolitan area after routine 10-valent pneumococcal conjugate vaccination of infants in Finland. *Eur. J. Clin. Microbiol. Infect. Dis.* 36 2109–2116. 10.1007/s10096-017-3033-5 28612153

[B45] Song J. Y. Nahm M. H. Moseley M. A. (2013). Clinical implications of pneumococcal serotypes: invasive disease potential, clinical presentations, and antibiotic resistance. *J. Korean Med. Sci.* 28 4–15. 10.3346/jkms.2013.28.1.4 23341706 PMC3546102

[B46] Sørensen U. B. (1993). Typing of pneumococci by using 12 pooled antisera. *J. Clin. Microbiol.* 31 2097–2100. 8370735 10.1128/jcm.31.8.2097-2100.1993 PMC265703

[B47] Spellerberg B. Brandt C. (2011). “Streptococcus,” in *Manual of Clinical Microbiology*, eds Versalovic J. Carroll K., G. Funke, J. Jorgensen, M. Landry, and D. Warnock (Washingtom, DC: ASM Press), 331–349.

[B48] Tan T. Q. (2012). Pediatric invasive pneumococcal disease in the United States in the era of pneumococcal conjugate vaccines. *Clin. Microbiol. Rev.* 25 409–419. 10.1128/CMR.00018-12 22763632 PMC3416489

[B49] Weinberger D. M. Malley R. Lipsitch M. (2011). Serotype replacement in disease after pneumococcal vaccination. *Lancet* 378 1962–1973. 10.1016/S0140-6736(10)62225-8 21492929 PMC3256741

[B50] Weiser J. N. (2010). The pneumococcus: why a commensal misbehaves. *J. Mol. Med.* 88 97–102. 10.1007/s00109-009-0557-x 19898768 PMC4487619

[B51] WHO (2012). Pneumococcal vaccines WHO position paper. *Vaccine* 30 4717–4718. 10.1016/j.vaccine.2012.04.093 22621828

[B52] WHO (2017). *Global Priority List of Antibiotic-Resistant Bacteria to Guide Research, Discovery, and Development of New Antibiotics.* Available at: http://www.who.int/medicines/publications/WHO-PPL-Short_Summary_25Feb-ET_NM_WHO.pdf

[B53] Zhou J. Y. Isaacson-Schmid M. Utterson E. C. Todd E. M. McFarland M. Sivapalan J. (2015). Prevalence of nasopharyngeal pneumococcal colonization in children and antimicrobial susceptibility profiles of carriage isolates. *Int. J. Infect. Dis.* 39 50–52. 10.1016/j.ijid.2015.08.010 26327122 PMC4620696

