# Supplementary material for: Evolution of Penicillin Non-susceptibility Among Streptococcus pneumoniae Isolates Recovered From Asymptomatic Carriage and Invasive Disease Over 25 years in Brazil, 1990–2014
Source: Front Microbiol. 2019 Mar 14;10:486. doi: 10.3389/fmicb.2019.00486 (PMC6427062; doi:10.3389/fmicb.2019.00486)
Supplement: Supplementary file 1 [file Table_1.DOCX]

Supplementary Material

Evolution of penicillin resistance among *Streptococcus pneumoniae* associated with asymptomatic carriage and invasive disease over 25 years in Brazil, 1990-2014

Tatiana Castro Abreu Pinto^1^, Felipe Piedade Gonçalves Neves^2^, Aline Rosa Vianna Souza^1^, Laura Maria Andrade Oliveira^1^, Natália Silva Costa^1^, Luciana Fundão Souza Castro^1^, [Cláudia Rezende de Vieira Mendonça-Souza](https://www.liebertpub.com/doi/abs/10.1089/mdr.2004.10.313?url_ver=Z39.88-2003&rfr_id=ori%3Arid%3Acrossref.org&rfr_dat=cr_pub%3Dpubmed&)^2^, José Mauro Peralta^1^, Lúcia Martins Teixeira^1*^

^1^Universidade Federal do Rio de Janeiro, Rio de Janeiro, Brazil

^2^Universidade Federal Fluminense, Niterói, Brazil

***Correspondence:**Lucia Martins Teixeira
lmt2@micro.ufrj.br

# Supplementary Table

Supplementary Table S1. Distribution of capsular types among 783 pneumococcal isolates, including 355 recovered from asymptomatic carriers (carriage column) and 428 from patients with invasive pneumococcal disease (IPD column) living in Brazil between 1990 and 2014.

| Capsular type^a^ | Carriage  Number (percentage) | IPD  Number (percentage) | Total number (%) of strains |
| --- | --- | --- | --- |
| 1 | None | 13 (3.0%) | 13 (1.7%) |
| 3 | 9 (2.5%) | 31 (7.2%) | 40 (5.1%) |
| **4** | **1 (0.3%)** | **20 (4.7%)** | **21 (2.7%)** |
| **5** | **3 (0.8%)** | **22 (5.1%)** | **25 (3.2%)** |
| **6A** | **19 (5.4%)** | **7 (1.6%)** | **26 (3.3%)** |
| **6B** | **35 (9.9%)** | **28 (6.5%)** | **63 (8.0%)** |
| **6C** | **32 (9.0%)** | **8 (1.9%)** | **40 (5.1%)** |
| 7B | 1 (0.3%) | None | 1 (0.1%) |
| 7C | 3 (0.8%) | 6 (1.4%) | 9 (1.1%) |
| 7F | None | 8 (1.9%) | 8 (1.0%) |
| 8 | None | 16 (3.7%) | 16 (2.0%) |
| 9A | 1 (0.3%) | 2 (0.5%) | 3 (0.4%) |
| 9L | None | 2 (0.5%) | 2 (0.2%) |
| **9N** | **1 (0.3%)** | **15 (3.5%)** | **16 (2.0%)** |
| **9V** | **9 (2.5%)** | **15 (3.5%)** | **24 (3.0%)** |
| **10A** | **7 (2.0%)** | **7 (1.6%)** | **14 (1.8%)** |
| 10F | None | 5 (1.2%) | 5 (0.6%) |
| **11A** | **4 (1.1%)** | **8 (1.9%)** | **12 (1.5%)** |
| 11B | None | 1 (0.2%) | 1 (0.1%) |
| 12A | None | 2 (0.5%) | 2 (0.2%) |
| 12F | 1 (0.3%) | 6 (1.4%) | 7 (0.9%) |
| 13 | 1 (0.3%) | 3 (0.7%) | 4 (0.5%) |
| **14** | **29 (8.2%)** | **57 (13.3%)** | **86 (11.0%)** |
| **15A/F** | **3 (0.8%)** | **5 (1.2%)** | **8 (1.0%)** |
| 15B | 9 (2.5%) | 1 (0.2%) | 10 (1.3%) |
| 15C | 15 (4.2%) | 2 (0.5%) | 17 (2.2%) |
| **16F** | **14 (4.0%)** | **10 (2.3%)** | **24 (3.1%)** |
| **17F** | **8 (2.3%)** | **6 (1.4%)** | **14 (1.8%)** |
| **18A** | **1 (0.3%)** | **6 (1.4%)** | **7 (0.9%)** |
| 18B | 2 (0.6%) | 3 (0.7%) | 5 (0.6%) |
| **18C** | **10 (2.8%)** | **11 (2.6%)** | **21 (2.7%)** |
| 18F | None | 3 (0.7%) | 3 (0.4%) |
| **19A** | **11 (3.1%)** | **11 (2.6%)** | **22 (2.8%)** |
| 19B | None | 1 (0.2%) | 1 (0.1%) |
| **19F** | **40 (11.3%)** | **22 (5.1%)** | **62 (7.9%)** |
| 20 | 5 (1.4%) | 5 (1.2%) | 10 (1.3%) |
| 21 | 1 (0.3%) | 3 (0.7%) | 4 (0.5%) |
| **22F** | **6 (1.7%)** | **1 (0.2%)** | **7 (0.9%)** |
| **23A** | **8 (2.3%)** | **3 (0.7%)** | **11 (1.4%)** |
| **23B** | **7 (2.0%)** | **2 (0.5%)** | **9 (1.1%)** |
| **23F** | **31 (8.7%)** | **20 (4.7%)** | **51 (6.5%)** |
| 24A | None | 1 (0.2%) | 1 (0.1%) |
| 24B | 1 (0.3%) | 1 (0.2%) | 2 (0.2%) |
| 24F | None | 3 (0.7%) | 3 (0.4%) |
| 25 | None | 1 (0.2%) | 1 (0.1%) |
| 28A | None | 1 (0.2%) | 1 (0.1%) |
| 28F | None | 5 (1.2%) | 5 (0.6%) |
| 29 | 1 (0.3%) | 2 (0.5%) | 3 (0.4%) |
| 31 | None | 1 (0.2%) | 1 (0.1%) |
| 33A | None | 1 (0.2%) | 1 (0.1%) |
| 33F | None | 2 (0.5%) | 2 (0.2%) |
| **34** | **2 (0.6%)** | **1 (0.2%)** | **3 (0.4%)** |
| **35A** | **None** | **2 (0.5%)** | **2 (0.2%)** |
| **35B** | **6 (1.7%)** | **1 (0.2%)** | **7 (0.9%)** |
| 35F | 1 (0.3%) | 1 (0.2%) | 2 (0.2%) |
| 36 | None | 1 (0.2%) | 1 (0.1%) |
| 37 | 3 (0.8%) | 1 (0.2%) | 4 (0.5%) |
| 38 | None | 3 (0.7%) | 3 (0.4%) |
| 39 | 1 (0.3%) | 1 (0.2%) | 2 (0.2%) |
| 40 | None | 2 (0.5%) | 2 (0.2%) |
| **NT** | **13 (3.7%)** | **None** | **13 (1.7%)** |

^a^NT, nontypeable. Capsular types included in the 10-valent pneumococcal conjugate vaccine are highlighted in green, and those only included in the 13-valent pneumococcal conjugate vaccine are highlighted in yellow. Capsular types comprising penicillin-nonsusceptible isolates are highlighted in bold.
